# Combination of Systemic Inflammation Response Index and Platelet-to-Lymphocyte Ratio as a Novel Prognostic Marker of Upper Tract Urothelial Carcinoma After Radical Nephroureterectomy

**DOI:** 10.3389/fonc.2019.00914

**Published:** 2019-09-18

**Authors:** Yangqin Zheng, Yuming Chen, Jingfeng Chen, Wu Chen, Yue Pan, Lianmin Bao, Xiaomin Gao

**Affiliations:** ^1^Department of Hematology, The Third Clinical Institute Affiliated to Wenzhou Medical University, People's Hospital of Wenzhou, Wenzhou, China; ^2^Department of Urology, Affiliated Hospital of Yangzhou University, Yangzhou, China; ^3^Department of Anorectal Surgery, Sixth Affiliated Hospital of Wenzhou Medical University (Lishui People's Hospital), Lishui, China; ^4^Department of Urology, The Third Clinical Institute Affiliated to Wenzhou Medical University, People's Hospital of Wenzhou, Wenzhou, China; ^5^Department of Urology, The First Affiliated Hospital of Wenzhou Medical University, Wenzhou, China; ^6^Department of Respiratory, Rui'an People's Hospital, The Third Affiliated Hospital of the Wenzhou Medical University, Wenzhou, China; ^7^Department of Urology, Changhai Hospital, Second Military Medical University, Shanghai, China

**Keywords:** upper tract urothelial carcinoma, systemic inflammation response index, platelet-to-lymphocyte ratio, prognosis, cancer

## Abstract

This study aimed to evaluate the preoperative prognostic value of systemic inflammation response index and platelet-to-lymphocyte ratio (SIRI-PLR) in patients with upper tract urothelial carcinoma (UTUC). The prognostic ability of SIRI-PLR was evaluated in a training cohort comprising 259 patients with UTUC who underwent radical nephroureterectomy and was further validated in an independent cohort comprising of 274 patients. Multivariate Cox regression models showed that SIRI was significantly associated with overall-survival (OS), cancer-specific survival (CSS), and metastatic-free survival (MFS), and PLR significantly affected OS and CSS (all *P* < 0.05). In particular, a simultaneously high SIRI-PLR value was considered an independent risk factor even after adjusting for confounding factors and was superior to SIRI alone in predicting survival among patients with UTUC. The analyses of concordance-index and receiver operating characteristic curve showed that incorporation of SIRI-PLR vs. without its incorporation into newly developed nomograms or currently available clinical parameters, such as pathologic T stage, N stage, or tumor grade, had higher accuracy in predicting urologic outcomes of patients with UTUC. These results were observed in the training cohort and were confirmed in the validation cohort. In conclusion, patients with a simultaneously high SIRI-PLR value had significantly poor prognosis. Incorporating SIRI-PLR into currently available clinical parameters can help in patient management.

## Introduction

Upper tract urothelial carcinomas (UTUCs) are relatively rare types of urologic cancer, and they account for ~5–10% of all urothelial carcinomas (1, 2). Because of their aggressive clinical and biological nature, ~60% of UTUCs are already invasive and 7% have metastasized at the time of diagnosis (3); hence, the prognosis is usually poor (4). Radical nephroureterectomy (RNU) with or without cisplatin-based combination chemotherapy remains the gold standard treatment for non-metastatic UTUC (5). However, almost 50% of patients who undergo RNU treatment experience recurrence (6). Moreover, the 5-year cancer-specific survival (CSS) rates are <50% for pathologic T2 and T3 (pT2/pT3) disease and <10% for pathologic T4 (pT4) disease (7–9). Thus, the evaluation of risk factors is important to identify patients who are more likely to experience disease recurrence after RNU.

Accumulating evidence has revealed the significant role of inflammatory markers in the development and progression of tumors. Previous studies have shown that inflammation biomarkers, including platelet-to-lymphocyte ratio (PLR), neutrophil-to-lymphocyte ratio (NLR), monocyte-to-lymphocyte ratio (MLR), and systemic inflammation response index (SIRI), are associated with worse urologic outcomes (10–13). However, the correlation between NLR and the prognosis of UTUC remains controversial (10). Several studies have shown that SIRI, which is based on neutrophil, monocyte, and lymphocyte count, is a significant risk factor for several cancers, including pancreatic adenocarcinoma (14), esophageal squamous cell carcinoma (15), gastric adenocarcinoma (16), renal cell carcinoma (11), thyroid carcinoma (17), and nasopharyngeal carcinoma (18). However, no study has reported the prognostic value of SIRI in postoperative recurrence and survival among patients with UTUC. Therefore, this study aimed to evaluate the prognostic value of SIRI, NLR, PLR, MLR, and combined use of inflammatory markers in patients who presented with UTUC after RNU.

## Patients and Methods

### Patients and Data Collection

Consecutive patients with UTUC (pathological T1-4N0-1M0) who underwent RNU at the First Affiliated Hospital of Wenzhou Medical University, China from March 2005 to August 2015 were retrospectively enrolled and included in the training cohort. Moreover, we retrospectively included an independent-validation cohort of consecutive patients who presented with UTUC (pathological T1-4N0-1M0) after RNU at The Third Clinical Institute Affiliated to Wenzhou Medical University, People's Hospital of Wenzhou, China from July 2003 to December 2016. The present study was approved by the ethics committees of The First Affiliated Hospital of Wenzhou Medical University and The Third Clinical Institute Affiliated to Wenzhou Medical University, People's Hospital of Wenzhou and was conducted according to the Declaration of Helsinki. All patients provided written informed consent.

**Figure 1:**
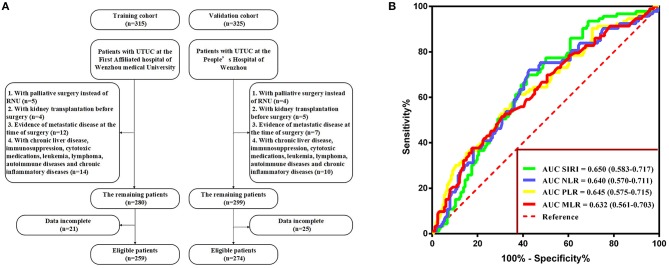
**(A)** Flow diagram of the study design. **(B)** Determination of the optimal cutoff value for SIRI, NLR, PLR, and MLR based on the ROC analysis.

The exclusion criteria were as follows: patients who underwent palliative surgery instead of RNU, those who underwent kidney transplantation before surgery, those with evidence of metastatic disease at the time of surgery, those with incomplete preoperative medical information on SIRI, NLR, PLR, MLR, and other clinical parameters, and those with relevant comorbidity affecting systemic inflammatory response markers (i.e., chronic liver disease, immunosuppression, cytotoxic medications, leukemia, lymphoma, autoimmune diseases, and chronic inflammatory diseases). None of the patients included in the study received preoperative adjuvant chemotherapy, radiotherapy, or other anti-tumor therapies. The selection participants are summarized in Figure 1A.

The following patient-specific information was obtained from our database for analysis: age, gender, grade according to the American Society of Anesthesiologists (ASA) physical status classification system, body mass index (BMI), hydronephrosis, surgical approach, NLR, PLR, MLR, SIRI, anemia, hypoproteinemia, chronic kidney disease (CKD) stage, tumor size, tumor site, multifocality, pathologic T stage, N stage, tumor grade, lymphovascular invasion (LVI), adjuvant therapy, and postoperative outcomes, including overall survival (OS), CSS, and metastatic-free survival (MFS).

Tumors were staged according to the American Joint Committee on Cancer TNM Classification, 7th edition, and tumor grading was assessed based on the World Health Organization (WHO) 2004 grading system. Tumor size was defined as the largest diameter of the tumor according to a pathological report. SIRI was defined as follows: SIRI = (neutrophil × monocyte)/lymphocyte. NLR was calculated as neutrophil count divided by lymphocyte count, PLR as platelet count divided by lymphocyte count, and MLR as monocyte count divided by lymphocyte count. Follow-up care comprised blood and urine tests, and chest and abdominal computed tomography scan or magnetic resonance imaging were performed every 3 months within the first year, then every 6 months from the second year to the fifth year, and annually thereafter. OS, CSS, and MFS were determined from the date of surgery to the date of death from any cause, date of cancer-specific death, and date of the last follow-up of recurrence of radiologically or histologically confirmed distant metastasis, respectively.

### Statistical Analysis

The cutoff values of SIRI, NLR, PLR, and MLR were determined using the Youden index by performing an analysis of the receiver operating characteristic (ROC) curves with OS as the endpoint. The comparisons between the clinicopathological characteristics of the patients and SIRI were performed using Student's *t*-test (normally distributed continuous variables), Mann-Whitney U test (non-normally distributed data), Pearson's chi-square test, or Fisher's exact test (categorical variables). The trends of SIRI for different PLR were analyzed using the Cochran-Armitage test. Survival patterns were identified using the means of the Kaplan-Meier curves. Univariate and multivariate Cox proportional hazards regression analyses were performed to evaluate the prognostic significance of each variable with respect to OS, CSS, and MFS. All *P*-values were two-tailed, and a *P* < 0.05 was considered statistically significant. Data were analyzed using SPSS (version, 25.0; IBM, Armonk, NY). Nomograms for the probability of OS, CSS, and MFS were established based on the results of the multivariate analysis using the R software (version 3.6.0) with rms, Hmisc, and ggplots packages. Calibration plot and concordance index (c-index) were used to evaluate the performance of the nomograms. A larger c-index represented a more accurate prognostic ability of the nomogram (low discriminative ability: 0.5–0.70, moderate discriminative ability: 0.71–0.90, and high discriminative ability: 0.90–1).

## Results

### Characteristics of the Participants

The clinicopathological characteristics of the two cohorts are summarized in Table 1. In the training cohort, 185 (71.4%) patients were men and 74 (28.6%) women, with a mean age of 67.5 ± 10.4 years. The median follow-up duration was 33.3 (interquartile range [IQR]: 15.5–64.2) months. During follow-up, 93 (35.9%) patients died, of whom 73 (28.2%) died of cancer-specific causes. Furthermore, 101 (39.0%) patients experienced distant metastasis. In the validation cohort, 184 (67.2%) patients were men and 90 (32.8%) women, with mean age of 65.9 ± 10.3 years. The median follow-up duration was 44.9 (IQR: 26.93–65.8) months. During follow-up, 85 (31.0%) patients died, whom 66 (24.1%) died of cancer-specific causes. In addition, 90 (32.8%) patients experienced distant metastasis.

**Table 1:** Characteristics of training and validation cohorts.

**Variables**	**Training cohort (*****n*** **=** **259)**			**Validation cohort (*****n*** **=** **274)**		
	**SIRI > 1.36** **(*n* = 122)**	**SIRI ≤ 1.36** **(*n* = 137)**	***P*-value**	**SIRI > 1.36** **(*n* = 117)**	**SIRI ≤ 1.36** **(*n* = 157)**	***P*-value**
Age (>65 vs. ≤65 years)	85/37	79/58	0.045^*^	69/48	91/66	0.866
Gender (Male vs. Female)	92/30	93/44	0.181	96/21	88/69	<0.001^*^
ASA grade (≥3 vs. <3)	34/88	27/110	0.122	19/98	27/130	0.834
BMI (≥25 vs. <25, Kg/m^2^)	14/108	37/100	0.002^*^	33/84	59/98	0.104
Hydronephrosis (Yes vs. No)	84/38	88/49	0.432	81/36	107/50	0.849
Surgical approach (laparoscopic vs. open)	36/86	53/84	0.121	102/15	145/12	0.155
NLR, Mean ± SD	4.93 ± 0.33	2.05 ± 0.06	<0.001^*^	4.74 ± 0.26	2.12 ± 0.09	<0.001^*^
PLR, Mean ± SD	195.65 ± 10.19	116.45 ± 3.56	<0.001^*^	162.69 ± 6.70	121.32 ± 4.53	<0.001^*^
MLR, Mean ± SD	0.52 ± 0.03	0.24 ± 0.08	<0.001^*^	0.48 ± 0.02	0.23 ± 0.01	<0.001^*^
Anemia (Yes vs. No)	67/55	41/96	<0.001^*^	38/79	42/115	0.302
Hypoproteinemia (Yes vs. No)	17/105	5/132	0.003^*^	14/103	10/147	0.105
CKD stage			0.008^*^			0.006^*^
CKD 1	7	21		35	29	
CKD 2	36	52		45	46	
CKD 3	61	55		31	73	
CKD 4	13	8		6	9	
CKD 5	5	1		0	0	
Tumor size (≥4 vs. <4cm)	30/92	23/114	0.120	34/83	22/135	0.002^*^
Tumor site			0.342			0.337
Pelvicalyceal	80	85		64	85	
Ureter	35	48		43	65	
Both	7	4		10	7	
Multifocality (Yes vs. No)	30/92	20/117	0.042^*^	29/88	37/120	0.815
Pathologic T stage			<0.001^*^			0.002^*^
pT1	29	51		32	60	
pT2	34	54		22	46	
pT3	38	24		49	45	
pT4	21	8		14	6	
N stage (N1 vs. N0)	18/104	6/131	0.004^*^	12/105	5/152	0.016^*^
Tumor grade (≥3 vs. <3)	94/28	106/31	0.951	92/25	105/52	0.032^*^
LVI (Yes vs. No)	28/94	13/124	0.003^*^	23/94	15/142	0.017^*^
Adjuvant therapy (Yes vs. No)	17/105	11/126	0.127	35/82	39/118	0.349
Follow-up duration, months, median (IQR)	27.50 (11.48–47.63)	39.70 (20.05–69.65)	<0.001^*^	32.90 (17.95–54.30)	52.00 (34.95–73.50)	<0.001^*^
All–cause death, *n* (%)	61 (50.00%)	32 (23.36%)	<0.001^*^	56 (47.86%)	29 (18.47%)	<0.001^*^
Cancer-specific death, *n* (%)	48 (39.34%)	25 (18.25%)	<0.001^*^	46 (39.32%)	20 (12.74%)	<0.001^*^
Metastasis, *n* (%)	59 (48.36%)	42 (30.66%)	<0.001^*^	53 (45.3%)	37 (23.57%)	<0.001^*^

* *Statistically significant*.

### Association Between SIRI and Clinicopathological Variables

With respect to OS, the optimal cutoff values were identified by ROC analysis and were as follows: SIRI, 1.36; NLR, 4.93; PLR, 195.65; and MLR, 0.52. The area under the curve (AUC) values of SIRI, NLR, PLR, and MLR were 0.650 (0.583–0.717), 0.640 (0.570–0.711), 0.645 (0.575–0.715), and 0.632 (0.561–0.703), respectively, with SIRI having the highest AUC (Figure 1B). The result indicated that SIRI was superior to the other three variables in terms of predicting survival.

The clinicopathological characteristics of the training and validation cohorts according to the cutoff value of SIRI are described in Table 1. In the training cohort, patients with higher SIRI were older than those with lower SIRI (*P* < 0.05). Lower BMI level, higher NLR, PLR, and MLR values, higher CKD stage, higher pathologic T stage, anemia, hypoproteinemia, multifocality, positive N status, and LVI were more commonly observed in patients with higher SIRI than in those with lower SIRI (all *P* < 0.05). In addition, patients with higher SIRI had a shorter follow-up duration, higher all-cause death, cancer-specific death, and were more likely to experience metastasis than those with lower SIRI (all *P* < 0.001). In the validation cohort, female sex, positive N status, LVI, higher NLR, PLR, and MLR values, higher CKD stage, larger tumor size, higher pathologic T stage, and higher tumor grade were more commonly observed in patients with higher SIRI than in those with lower SIRI (all *P* < 0.05). In addition, patients with higher SIRI had a shorter follow-up duration, higher all-cause death, cancer-specific death, and were more likely to experience metastasis than those with lower SIRI (all *P* values < 0.001).

### Prognostic Significance of SIRI, NLR, PLR, and MLR

**Figure 2:**
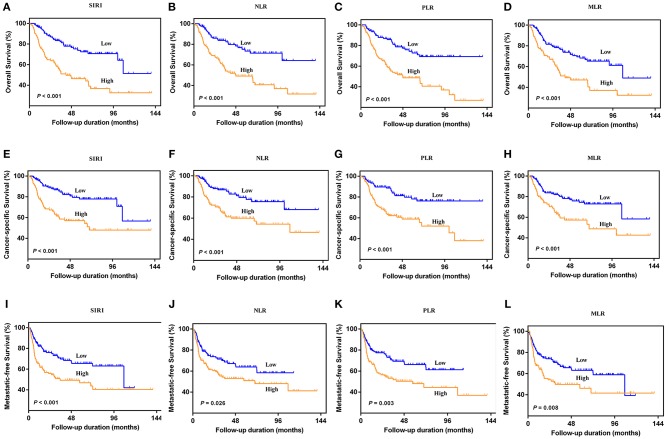
Kaplan-Meier curves for OS **(A–D)**, CSS **(E–H)**, and MFS **(I–L)** in UTUC patients stratified by SIRI, NLR, PLR, and MLR in the training cohort.

The Kaplan-Meier survival curves showed lower OS, CSS, and MFS in patients with higher SIRI, NLR, PLR, and MLR values both in the training and validation cohorts (all *P* < 0.05) (Figure 2 and Figure S1). The multivariate Cox regression model showed that SIRI was an independent risk factor of OS (hazard ratio [HR] = 2.005, 95%CI, 1.103–3.644, *P* = 0.022), CSS (HR = 2.271, 95%CI, 1.146–4.500, *P* = 0.019), and MFS (HR = 2.257, 95%CI, 1.277–3.987, *P* = 0.005) in the training cohort. Similar findings were observed in the validation cohort (OS: HR = 2.797, 95%CI, 1.249–6.262, *P* = 0.012; CSS: HR = 3.096, 95%CI, 1.262–7.599, *P* = 0.014; MFS: HR = 1.906, 95%CI, 0.931–3.905, *P* = 0.048). PLR was considered a significant risk factor for OS in the training cohort (HR = 1.786, 95%CI, 1.020–3.128, *P* = 0.043). However, in the validation cohort, PLR was a significant risk factor for both OS (HR = 1.839, 95%CI, 1.040–3.253, *P* = 0.036) and CSS (HR = 1.951, 95%CI, 1.047–3.634, *P* = 0.035). Other risk predictors included age, hydronephrosis, tumor size, pathologic T stage, N stage, LVI, adjuvant therapy, and tumor site (all *P* < 0.05) (Tables 2, 3 and Tables S1, S2).

**Table 2:** Univariate analysis of variables for the prediction of survival outcomes in training cohort.

**Variables**	**Overall survival**			**Cancer-specific survival**			**Metastasis-free survival**		
	**HR**	**95%CI**	***P* value**	**HR**	**95%CI**	***P* value**	**HR**	**95%CI**	***P* value**
Gender (Male vs. Female)	0.887	0.569–1.382	0.595	0.892	0.541–1.471	0.655	1.062	0.687–1.643	0.786
Age (>65 vs. ≤65 years)	2.025	1.276–3.214	0.003^*^	1.780	1.069–2.966	0.027^*^	1.526	1.002–2.325	0.049^*^
BMI (≥25 vs. <25)	0.414	0.089–0.680	0.007^*^	0.417	0.191–0.909	0.028^*^	0.434	0.232–0.812	0.009^*^
ASA grade (≥3 vs. <3)	1.541	0.993–2.391	0.054	1.355	0.816–2.251	0.241	1.155	0.739–1.804	0.528
Hydronephrosis (Yes vs. No)	1.543	0.970–2.457	0.067	1.895	1.088–3.300	0.024^*^	1.991	1.241–3.195	0.004^*^
Surgical approach (laparoscopic vs. open)	0.696	0.431–1.125	0.140	0.722	0.423–1.231	0.232	0.707	0.452–1.106	0.129
SIRI (≥1.36 vs. <1.36)	2.298	1.515–3.487	<0.001^*^	2.736	1.684–4.445	<0.001^*^	1.950	1.311–2.901	0.001^*^
NLR (≥2.53 vs. <2.53)	2.718	1.726–4.280	<0.001^*^	2.385	1.445–3.937	0.001^*^	1.573	1.053–2.350	0.027^*^
PLR (≥126.88 vs. <126.88)	2.780	1.780–4.341	<0.001^*^	2.673	1.617–4.419	**<0.001**	1.844	1.231–2.763	0.003^*^
MLR (≥0.35 vs. <0.35)	2.162	1.436–3.254	<0.001^*^	2.141	1.350–3.397	0.001^*^	1.692	1.142–2.507	0.009^*^
Anemia (Yes vs. No)	1.913	1.270–2.882	0.002^*^	0.262	0.063–1.089	0.065	1.791	1.210–2.650	0.004^*^
Hypoproteinemia (Yes vs. No)	2.618	1.476–4.643	0.001^*^	1.802	1.136–2.859	0.012^*^	1.907	1.063–3.421	0.030^*^
CKD stage									
CKD 1	1.000	Reference	1.000	1.000	Reference	1.000	1.000	Reference	1.000
CKD 2–3	2.888	1.054–7.911	0.039^*^	2.931	0.917–9.370	0.070	2.486	1.006–6.141	0.048^*^
CKD 4–5	6.039	2.007–18.176	0.001^*^	6.097	1.728–21.514	0.005^*^	4.753	1.734–13.025	0.002^*^
Tumor size (≥3 vs. <3)	1.600	1.061–2.412	0.025^*^	1.061	0.971–1.159	0.190	1.564	1.056–2.318	0.026^*^
Tumor site									
Pelvicalyceal	1.000	Reference	1.000	1.000	Reference	1.000	1.000	Reference	1.000
Ureter	1.405	0.910–2.170	0.125	1.606	0.988–2.613	0.056	1.576	1.042–2.384	0.031^*^
Both	1.701	0.679–4.259	0.257	2.345	0.923–5.959	0.073	2.232	1.106–4.905	0.046^*^
Multifocality (Yes vs. No)	1.640	1.023–2.628	0.040^*^	1.749	1.036–2.952	0.036^*^	1.380	0.866–2.198	0.175
Pathologic T stage									
pT1	1.000	Reference	1.000	1.000	Reference	1.000	1.000	Reference	1.000
pT2 vs. pT1	1.352	0.695–2.630	0.374	2.241	0.918–5.470	0.076	1.982	1.083–3.630	0.027^*^
pT3 vs. pT1	4.108	2.221–7.597	<0.001^*^	6.988	3.029–16.125	<0.001^*^	3.391	1.840–6.247	<0.001^*^
pT4 vs. pT1	13.121	6.761–25.464	<0.001^*^	25.422	10.709–60.346	<0.001^*^	9.626	5.056–18.329	<0.001^*^
N stage (N1 vs. N0)	7.359	4.435–12.211	<0.001^*^	8.945	5.235–15.283	<0.001^*^	5.291	3.240–8.641	<0.001^*^
Tumor grade (≥3 vs. <3)	2.641	1.277–5.463	0.009^*^	4.443	1.619–12.191	0.004^*^	2.244	1.227–4.104	0.009^*^
LVI (Yes vs. No)	4.830	3.116–7.485	<0.001^*^	6.248	4.004–10.321	<0.001^*^	3.848	2.508–5.905	<0.001^*^
Adjuvant therapy (Yes vs. No)	2.842	1.746–4.625	<0.001^*^	3.614	2.158–6.053	<0.001^*^	3.543	2.214–5.672	<0.001^*^

* *Statistically significant*.

**Table 3:** Multivariate analysis of variables for the prediction of survival outcomes in training cohort.

**Variables**	**Overall survival**			**Cancer-specific survival**			**Metastasis-free survival**		
	**HR**	**95%CI**	***P* value**	**HR**	**95%CI**	***P* value**	**HR**	**95%CI**	***P* value**
Age (>65 vs. ≤65 years)	2.119	1.234–3.636	0.006^*^	1.746	0.941–3.240	0.077	1.521	0.932–2.482	0.094
BMI (≥25 vs. <25)	0.795	0.367–1.726	0.562	0.948	0.388–2.314	0.907	0.578	0.284–1.173	0.129
ASA grade (≥3 vs. <3)	1.593	0.916–2.769	0.099		–			–	
Hydronephrosis (Yes vs. No)	2.009	1.204–3.353	0.008^*^	1.940	0.958–3.928	0.066	1.855	1.059–3.248	0.031^*^
SIRI (≥1.36 vs. <1.36)	2.005	1.103–3.644	0.022^*^	2.271	1.146–4.500	0.019^*^	2.257	1.277–3.987	0.005^*^
NLR (≥2.53 vs. <2.53)	0.855	0.435–1.683	0.651	0.687	0.314–1.504	0.348	0.459	0.240–0.876	0.118
PLR (≥126.88 vs. <126.88)	1.786	1.020–3.128	0.043^*^	1.650	0.867–3.142	0.127	1.333	0.791–2.247	0.280
MLR (≥0.35 vs. <0.35)	0.708	0.408–1.229	0.220	0.759	0.402–1.432	0.395	0.901	0.525–1.548	0.706
Anemia (Yes vs. No)	0.979	0.585–1.638	0.934	0.923	0.499–1.709	0.799	1.314	0.816–2.116	0.261
Hypoproteinemia (Yes vs. No)	0.785	0.374–1.646	0.522	0.878	0.380–2.025	0.760	0.784	0.370–1.660	0.525
CKD stage									
CKD 1	1.000	Reference	1.000	1.000	Reference	1.000	1.000	Reference	1.000
CKD 2–3	1.372	0.464–4.052	0.567	1.323	0.377–4.644	0.662	1.025	0.383–2.743	0.961
CKD 4–5	1.284	0.372–4.431	0.693	1.357	0.322–5.723	0.678	1.300	0.399–4.235	0.663
Tumor size (≥3 vs. <3)	1.682	1.060–2.669	0.027^*^	2.010	1.168–3.460	0.012^*^	1.526	0.992–2.348	0.055
Tumor site									
Pelvicalyceal		–		1.000	Reference	1.000	1.000	Reference	1.000
Ureter				1.928	1.032–3.603	0.040^*^	1.656	0.982–2.794	0.059
Both				1.914	0.646–5.674	0.242	1.508	0.633–3.591	0.354
Multifocality (Yes vs. No)	1.043	0.600–1.813	0.880	1.236	0.668–2.287	0.500		–	
Pathologic T stage									
pT1	1.000	Reference	1.000	1.000	Reference	1.000	1.000	Reference	1.000
pT2 vs. pT1	1.313	0.649–2.655	0.449	1.795	0.699–4.609	0.224	1.837	0.963–3.503	0.065
pT3 vs. pT1	2.812	1.394–5.675	0.004^*^	4.202	1.658–10.651	0.002^*^	2.181	1.089–4.368	0.028^*^
pT4 vs. pT1	6.586	1.536–28.239	0.011^*^	17.249	3.435–86.610	0.001^*^	6.585	1.590–27.267	0.009^*^
N stage (N1 vs. N0)	1.215	0.239–6.189	0.014^*^	1.518	1.098–2.732	0.038^*^	1.124	0.219–5.761	0.888
Tumor grade (≥3 vs. <3)	1.370	0.626–2.997	0.430	2.216	0.750–6.547	0.150	1.484	0.773–2.850	0.235
LVI (Yes vs. No)	1.730	0.776–3.856	0.180	2.831	1.179–6.793	0.020^*^	1.281	0.560–2.931	0.558
Adjuvant therapy (Yes vs. No)	1.905	1.114–3.257	0.019^*^	2.161	1.222–3.820	0.008^*^	2.345	1.402–3.924	0.001^*^

* *Statistically significant*.

### Association Between Survival and SIRI-PLR

Previous studies have shown that UTUC patients with elevated preoperative PLR had significantly worse survival outcomes (13, 19, 20). In addition, SIRI consists of neutrophil, monocyte, and lymphocyte count, but not platelet count. Therefore, we further evaluated the prognostic value of SIRI-PLR.

**Figure 3:**
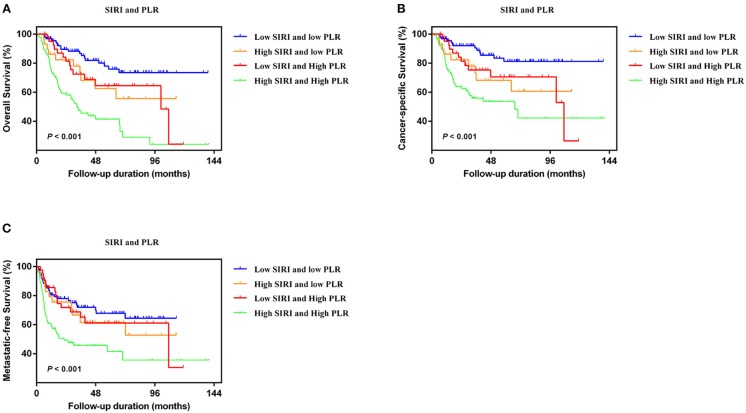
Kaplan-Meier analysis for OS **(A)**, CSS **(B)**, and MFS **(C)** in patients with UTUC who was divided into four groups based on SIRI-PLR in the training cohort.

In the two independent cohorts, PLR was positively correlated to SIRI (Figure S2) (training cohort: *r* = 0.455, *P* < 0.001; validation cohort: *r* = 0.320, *P* < 0.001). Subsequently, patients were categorized into four groups: patients with low SIRI and low PLR, patients with high SIRI and low PLR, patients with low SIRI and high PLR, and patients with high SIRI and high PLR. The Kaplan-Meier curves showed that patients with high SIRI and high PLR had the lowest OS, CSS, and MFS (all *P* < 0.001) (Figure 3 and Figure S3). The multivariate analysis revealed that the combination of high SIRI and high PLR was a significant risk predictor for OS, CSS, and MFS in both the training cohort (OS: HR = 2.405, 95%CI, 1.288–4.490, *P* = 0.006; CSS: HR = 2.351, 95%CI, 1.141–4.844, *P* = 0.021; MFS: HR = 2.352, 95%CI, 1.283–4.333, *P* = 0.027) and the validation cohort (OS: HR = 3.829, 95%CI, 1.999–7.335, *P* < 0.001; CSS: HR = 4.322, 95%CI, 2.038-9.164, *P* < 0.001; and MFS: HR = 2.565, 95%CI, 1.388–4.739, *P* = 0.003) (Table 4 and Table S3). Figure S4 shows that SIRI-PLR was superior to SIRI alone in predicting survival. Therefore, high SIRI-PLR was considered an independent risk predictor of OS, CSS, and MFS in patients with UTUC.

**Table 4:** Multivariate analysis of variables for the prediction of survival outcomes in training cohort when interrelated SIRI and PLR are combined.

**Variables**	**Overall survival**			**Cancer-specific survival**			**Metastasis-free survival**		
	**HR**	**95%CI**	***P* value**	**HR**	**95%CI**	***P* value**	**HR**	**95%CI**	***P* value**
Age (>65 vs. ≤ 65 years)	2.202	1.274–3.792	0.004^*^	1.696	0.919–3.129	0.091	1.563	0.955–2.559	0.076
BMI (≥25 vs. <25)	0.856	0.396–1.853	0.694	1.012	0.416–2.459	0.980	0.670	0.333–1.345	0.260
ASA grade (≥3 vs. <3)	1.425	0.822–2.471	0.207		–				
Hydronephrosis (Yes vs. No)	2.048	1.234–3.399	0.006^*^	1.952	0.969–3.932	0.061	1.936	1.110–3.374	0.020^*^
SIRI-PLR									
Low SIRI + low PLR	1.000	Reference	1.000	1.000	Reference	1.000	1.000	Reference	1.000
High SIRI + low PLR vs. low SIRI + low PLR	1.087	0.473–2.499	0.844	1.388	0.546–3.530	0.491	0.806	0.369–1.760	0.588
Low SIRI + high PLR vs. low SIRI + low PLR	1.134	0.514–2.500	0.756	1.201	0.492–2.932	0.688	0.605	0.289–1.269	0.184
High SIRI + high PLR vs. low SIRI + low PLR	2.405	1.288–4.490	0.006^*^	2.351	1.141–4.844	0.021^*^	2.352	1.283–4.333	0.027^*^
Anemia (Yes vs. No)	0.902	0.548–1.484	0.685	0.809	0.455–1.441	0.472	1.230	0.772–1.958	0.384
Hypoproteinemia (Yes vs. No)	0.843	0.410–1.733	0.642	0.906	0.401–2.048	0.812	0.771	0.370–1.608	0.488
CKD stage									
CKD 1	1.000	Reference	1.000	1.000	Reference	1.000	1.000	Reference	1.000
CKD 2–3	1.370	0.468–4.011	0.566	1.376	0.393–4.818	0.618	1.049	0.397–2.770	0.923
CKD 4–5	1.267	0.370–4.336	0.706	1.358	0.324–5.694	0.675	1.279	0.401–4.075	0.678
Tumor size (≥3 vs. <3)	1.784	1.126–2.828	0.014^*^	2.009	1.179–3.422	0.010^*^	1.539	1.000–2.369	0.050
Tumor site									
Pelvicalyceal				1.000	Reference	1.000	1.000	Reference	1.000
Ureter				1.917	1.031–3.566	0.040^*^	1.574	0.937–2.643	0.086
Both				1.968	0.661–5.860	0.224	1.642	0.686–3.930	0.265
Multifocality (Yes vs. No)	1.015	0.583–1.769	0.957	1.174	0.637–2.163	0.607			
Pathologic T stage									
pT1	1.000	Reference	1.000	1.000	Reference	1.000	1.000	Reference	1.000
pT2 vs. pT1	1.210	0.597–2.450	0.597	1.694	0.661–4.342	0.273	1.568	0.826–2.980	0.169
pT3 vs. pT1	2.708	1.347–5.446	0.005^*^	4.184	1.664–10.523	0.002^*^	2.164	1.081–4.333	0.029^*^
pT4 vs. pT1	5.836	1.389–24.515	0.016^*^	15.633	3.200–76.369	0.001^*^	6.152	1.521–24.872	0.011^*^
N stage (N1 vs. N0)	1.306	0.265–6.445	0.043^*^	1.552	1.110–3.783	0.034^*^	1.040	0.211–5.114	0.042^*^
Tumor grade (≥3 vs. <3)	1.366	0.624–2.991	0.436	2.289	0.771–6.796	0.136	1.351	0.699–2.610	0.371
LVI (Yes vs. No)	1.627	0.741–3.574	0.225	2.568	1.106–5.967	0.028^*^	1.251	0.563–2.781	0.582
Adjuvant therapy (Yes vs. No)	1.993	1.160–3.424	0.013^*^	2.288	1.282–4.086	0.005^*^	2.564	1.504–4.370	0.001^*^

* *Statistically significant*.

### Nomogram and Its Performance

**Figure 4:**
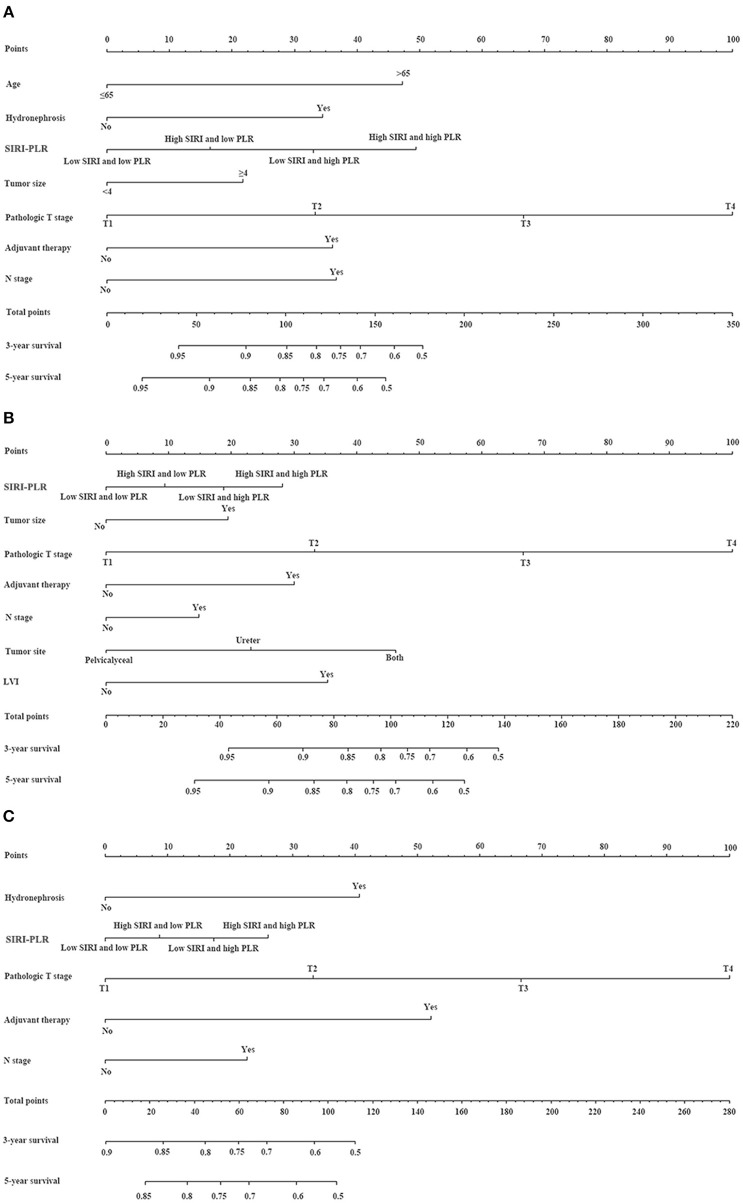
Establishment of nomograms for the prediction of OS **(A)**, CSS **(B)**, and MFS **(C)** in patients with UTUC after surgery. To use the nomogram, the value of individual patients with UTUC is shown on each variable axis, and a line is depicted upward to determine the number of points received for each variable value. Subsequently, the sum of these numbers is located on the total point axis, and a line is drawn downward to the survival axes to determine the likelihood of 3- and 5-year survival of OS, CSS, and MFS.

**Figure 5:**
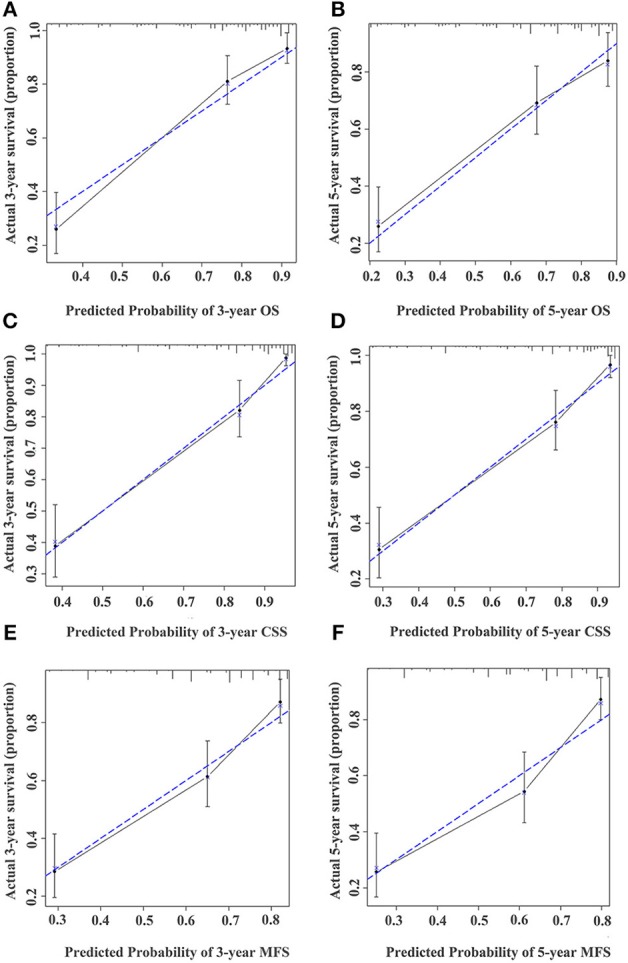
Calibration curve for predicting the 3- and 5-year survival of OS **(A,B)**, CSS **(C,D)**, and MFS **(E,F)** in UTUC patients in the training cohort. The actual OS, CSS, and MFS rates are plotted on the y-axis and nomogram-predicted OS, CSS, and MFS rates are plotted on the x-axis.

**Figure 6:**
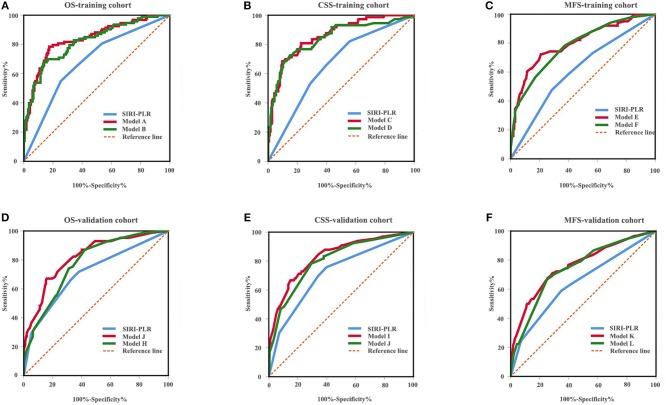
ROC analysis of the prognostic accuracy of SIRI-PLR for OS **(A,D)**, CSS **(B,E)**, and MFS **(C,F)** in training cohort and validation cohort.

We developed prognostic nomograms for OS, CSS, and MFS (Figure 4 and Figure S5) using independent predictors identified in the multivariate Cox regression models. A score was assigned to each predictor in the nomogram (top scale). The sum of these scores represented the probability of 3- and 5-year urological survival (bottom scale). The calibration plots of these nomograms were developed (Figure 5 and Figure S6), which showed that the nomograms were well-calibrated. In the training cohort, by incorporating the SIRI-PLR into the models, the c-indexes for the nomograms of OS, CSS, and MFS increased from 0.795 (0.748–0.842) to 0.808 (0.767–0.859), from 0.827 (0.785–0.859) to 0.844 (0.805–0.883), and from 0.733 (0.685–0.781) to 0.747 (0.699–0.795), respectively, indicating that this new biomarker can improve the prognostic accuracy in patients with UTUC. Similar results were observed in the validation cohort (Table 5 and Figure S7). In addition, the c-index value of SIRI-PLR combined with pT, N stage, LVI, or tumor grade for OS, CSS, or MFS in both cohorts was higher than that of SIRI-PLR or any indicator alone (Table 5). By incorporating SIRI-PLR into the models, the AUC and performance of the other indicators, which were associated with the prediction performance of nomograms for OS, CSS, and MFS, also improved (Table 6 and Figure 6). Therefore, combining SIRI-PLR and the currently available clinical parameters may help in patient risk stratification and clinical decision-making.

**Table 5:** C-index analysis of the prognostic accuracy of SIRI-PLR and other variables for OS, CSS, and MFS in training and validation cohorts.

**Characteristics**	**OS**	**CSS**	**MFS**
**Training cohort**			
Age	0.566 (0.515–0.617)	–	–
Hydronephrosis	0.553 (0.506–0.600)	–	0.570 (0.525–0.615)
SIRI-PLR	0.668 (0.615–0.721)	0.665 (0.606–0.724)	0.610 (0.554–0.666)
Tumor size	0.568 (0.514–0.622)	0.569 (0.509–0.629)	–
Tumor site	–	0.562 (0.500–0.624)	–
pT	0.744 (0.963–0.795)	0.787 (0.739–0.835)	0.696 (0.644–0.748)
N stage	0.620 (0.574–0.666)	0.639 (0.586–0.685)	0.601 (0.562–0.640)
LVI	–	0.679 (0.624–0.734)	–
Adjuvant therapy	0.564 (0.509–0.604)	0.585 (0.537–0.633)	0.575 (0.538–0.612)
SIRI-PLR + pT	0.779 (0.735–0.823)	0.812 (0.767–0.857)	0.713 (0.660–0.766)
SIRI-PLR + N stage	0.742 (0.693–0.791)	0.755 (0.701–0.809)	0.666 (0.610–0.722)
SIRI-PLR + LVI	–	0.781 (0.730–0.832)	–
Model A	0.808 (0.767–0.859)		
Model B	0.795 (0.748–0.842)		
Model C		0.844 (0.805–0.883)	
Model D		0.827 (0.785–0.859)	
Model E			0.747 (0.699–0.795)
Model F			0.733 (0.685–0.781)
**Validation cohort**			
SIRI-PLR	0.689 (0.635–0.743)	0.708 (0.649–0.762)	0.642 (0.586–0.698)
Tumor site	0.518 (0.456–0.580)	–	–
pT	0.740 (0.688–0.792)	0.773 (0.719–0.827)	0.721 (0.669–0.773)
N stage	0.611 (0.571–0.652)	0.625 (0.581–0.669)	0.605 (0.573–0.637)
Adjuvant therapy	–	0.582 (0.521–0.643)	–
Tumor grade	–	–	0.601 (0.562–0.640)
SIRI-PLR + pT	0.768 (0.718–0.818)	0.806 (0.753–0.859)	0.741 (0.687–0.795)
SIRI-PLR + N stage	0.705 (0.652–0.758)	0.718 (0.659–0.777)	0.665 (0.608–0.722)
SIRI-PLR + Tumor grade	–	–	0.687 (0.632–0.742)
Model G	0.778 (0.727–0.829)		
Model H	0.759 (0.705–0.813)		
Model I		0.815 (0.762–0.868)	
Model J		0.795 (0.739–0.851)	
Model K			0.750 (0.696–0.804)
Model L			0.731 (0.679–0.783)

*Model A = age + hydronephrosis + SIRI-PLR + tumor size + pT + N stage + adjuvant therapy*.

*Model B = age + hydronephrosis + tumor size + pT + N stage + adjuvant therapy*.

*Model C = SIRI-PLR + tumor size + tumor site + pT + N stage + LVI + adjuvant therapy*.

*Model D = tumor size + tumor site + pT + N stage + LVI + adjuvant therapy*.

*Model E = hydronephrosis + SIRI-PLR + pT + N stage + adjuvant therapy*.

*Model F = hydronephrosis + pT + N stage + adjuvant therapy*.

*Model G = SIRI-PLR + tumor site + pT + N stage*.

*Model H = tumor site + pT + N stage*.

**Table 6:** ROC analysis of the prognostic accuracy of SIRI-PLR for OS, CSS, and MFS in training and validation cohorts.

**Model**	**AUC (95% CI)**	**Sensitivity %**	**Specificity %**	**Youden index**	**Positive likelihood ratio**	**Negative likelihood ratio**
**Training cohort**						
OS						
Model A	0.836 (0.784–0.889)	78.49	82.53	0.610	4.49	0.26
Model B	0.819 (0.765–0.874)	67.74	86.14	0.539	4.89	0.37
SIRI-PLR	0.681 (0.613–0.748)	54.84	74.70	0.295	2.17	0.60
CSS						
Model C	0.858 (0.808–0.908)	68.49	90.32	0.588	7.08	0.35
Model D	0.842 (0.783–0.900)	72.60	84.95	0.576	4.82	0.32
SIRI-PLR	0.663 (0.591–0.734)	82.19	36.83	0.263	1.47	0.48
MFS						
Model E	0.799 (0.742–0.856)	68.32	86.54	0.519	4.15	0.37
Model F	0.782 (0.724–0.839)	78.22	62.66	0.409	2.10	0.35
SIRI-PLR	0.613 (0.543–0.684)	47.52	71.52	0.190	1.67	0.73
**Validation cohort**						
OS						
Model G	0.819 (0.764–0.873)	67.06	84.13	0.512	4.23	0.39
Model H	0.777 (0.720–0.833)	87.06	57.67	0.447	2.06	0.22
SIRI-PLR	0.708 (0.639–0.777)	71.76	61.90	0.334	1.88	0.46
CSS						
Model I	0.830 (0.771–0.889)	66.67	85.10	0.518	4.47	0.39
Model J	0.802 (0.740–0.864)	78.79	70.19	0.490	2.64	0.30
SIRI-PLR	0.716 (0.642–0.789)	75.76	60.10	0.359	1.90	0.40
MFS						
Model K	0.761 (0.699–0.824)	71.11	71.20	0.423	2.47	0.41
Model L	0.742 (0.679–0.805)	66.67	75.00	0.417	2.67	0.44
SIRI-PLR	0.643 (0.572–0.715)	58.89	65.22	0.241	1.69	0.63

*OS, overall survival; CSS, cancer-specific survival; MFS, metastatic-free survival; SIRI-PLR, systemic inflammation response index- platelet-to-lymphocyte ratio; AUC, area under the curve*.

*Model A = age + hydronephrosis + SIRI-PLR + tumor size + pT + N stage + adjuvant therapy*.

*Model B = age + hydronephrosis + tumor size + pT + N stage + adjuvant therapy*.

*Model C = SIRI-PLR + tumor size + tumor site + pT + N stage + LVI + adjuvant therapy*.

*Model D = tumor size + tumor site + pT + N stage + LVI + adjuvant therapy*.

*Model E = hydronephrosis + SIRI-PLR + pT + N stage + adjuvant therapy*.

*Model F = hydronephrosis + pT + N stage + adjuvant therapy*.

*Model G = SIRI-PLR + tumor site + pT + N stage*.

*Model H = tumor site + pT + N stage*.

*Model I = SIRI-PLR + pT + N stage + adjuvant therapy*.

*Model J = pT + N stage + adjuvant therapy*.

*Model K = SIRI-PLR + pT + N stage + tumor grade*.

*Model L = pT + N stage + tumor grade*.

## Discussion

In the present study, the prognostic value of SIRI was evaluated in a training cohort and was confirmed in a validation cohort. The ROC analysis showed that the cutoff value of SIRI was 1.36, and SIRI values higher than 1.36 were significantly associated with high pT stage, positive N status, positive LVI, high CKD stage, and other clinical parameters indicative of an aggressive phenotype. Multivariate Cox regression models showed that SIRI was an independent predictor of OS, CSS, and MFS, and PLR was also significantly associated with lower OS or CSS. Furthermore, a positive correlation was observed between PLR and SIRI. Moreover, SIRI-PLR was a significant risk factor of lower OS, CSS, and MFS and was superior than SIRI or PLR alone in predicting survival. When we further incorporated SIRI-PLR into the models or currently available clinical parameters, the prognostic accuracy of OS, CSS, and MFS improved. To the best of our knowledge, this report first showed that SIRI-PLR can be a significant indicator in predicting the prognosis of patients with UTUC; hence, it can be applied during risk stratification and clinical decision-making.

Increasing evidence has consistently shown that systemic inflammation could contribute to the growth, deterioration, and metastasis of cancer (21), thereby affecting impact survival patterns. Inflammatory processes involving cytokines, small inflammatory proteins, and immune cells are considered a hallmark of cancer (22). Inflammation-based factors and systemic inflammatory scores, including NLR, PLR, MLR, and systemic immune-inflammation index (SII), are considered independent markers of prognosis and can further improve the prognostic accuracy of the models established for multiple malignant tumors (10–12, 20, 23). SIRI, a novel inflammatory related marker, is significantly associated with postoperative recurrence and metastasis in patients with several types of carcinomas (11, 14–16, 18). Furthermore, numerous studies have shown that the predictive ability of SIRI is more powerful than that of other inflammatory factors, such as NLR, PLR, and MLR (11, 14, 15). Our findings are consistent with those of previous studies as only SIRI and PLR were considered as independent predictors.

There is uncertainty as to why high SIRI-PLR increases the risk of tumor recurrence and mortality, although this result might be explained by the functions of neutrophil, lymphocyte, monocyte, and platelet. Neutrophils may create an inflammatory microenvironment by producing anti-microbial and immunoregulatory mediators, resulting in tumor development, angiogenesis, progression, and metastasis and protecting tumor cells from immune surveillance (24, 25). Moreover, monocytes and monocyte-derived macrophages play an important role in tumor growth, invasion, and suppression of antitumor immunity and dissemination (26, 27). To some extent, monocyte count can represent a patient's tumor burden (28). Platelets facilitate tumor progression and metastasis (29, 30) and may also have other functions correlated to the generation of macrophages and neutrophils by recruiting and regulating monocytic and granulocytic cells (27). In contrast, lymphocytes enhance the anti-tumor efforts by secreting cytokines, such as interferon gamma (INF-γ) and tumor necrosis factor (TNF-α), thereby promoting cytotoxic cell death (25). The immune response to cancer mainly relies on the peripheral blood level of lymphocytes; however, they can be rapidly decreased due to systemic inflammation (11). For example, the activation of T cells can be impaired by increased circulating neutrophils attributed to the secretion of large amounts of nitric oxide, arginase, and reactive oxygen species (31). Accordingly, the crosstalk and cooperation between these inflammatory cells and related inflammatory mediators (i.e., chemokines and cytokines.) in the microenvironment of tumor inflammation may contribute to tumorigenesis and cancer progression. Therefore, our findings revealed that SIRI-PLR is an objective and reliable marker that reflects the tumor burden, and a simultaneously high circulating SIRI-PLR levels may be highly indicative of immune escape and increased circulating tumor cell levels, which may ultimately lead to poor urologic outcomes. These findings may be important for urologists in terms of clinical decision-making process and in particular identifying patients qualified for aggressive therapy.

Our study had several strengths. First, the predictive value of SIRI-PLR was confirmed in an independent cohort. Second, SIRI-PLR was first introduced in UTUC for evaluation, and this new biomarker was found to have a more powerful prognostic ability than NLR, PLR, MLR, and SIRI. Third, SIRI-PLR is advantageous as it is non-invasive, easy to assess, highly reproducible, affordable, and, more importantly, feasible, with a high accuracy in predicting the survival of patients who present with UTUC after RNU.

However, the current study also had some limitations. First, this study utilized data collected retrospectively, which may result in potential errors or misclassifications. However, all results were validated using data from another independent cohort. Moreover, although we adjusted multiple covariates in our multivariate Cox regression models, residual confounding cannot be ruled out. Hence, randomized controlled trials must be conducted to validate the prognostic ability of SIRI-PLR. Second, this study could not establish an optimal cutoff value for SIRI as the cutoff values for the two cohorts were inconsistent: 1.36 in the training cohort and 1.48 in the validation cohort. Thus, the lack of a consistent cutoff level might have influenced the prognostic ability of SIRI-PLR in different settings. Therefore, additional studies and meta-analysis should be further performed to establish the optimal cutoff values of SIRI. Third, we could not evaluate the effects of dynamic changes in SIRI-PLR on survival because of incomplete data. Thus, these effects must be further evaluated in future studies.

## Conclusions

The combination of SIRI-PLR is a non-invasive, easily accessible, and highly accurate predictor of prognosis among patients who present with UTUC after RNU. Urologists should consider the assessment results of this novel inflammation-based biomarker during clinical decision-making.

## Data Availability

All datasets generated for this study are included in the manuscript/Supplementary Files.

## Ethics Statement

The studies involving human participants were reviewed and approved by the ethics committees of The First Affiliated Hospital of Wenzhou Medical University and The Third Clinical Institute Affiliated to Wenzhou Medical University, People's Hospital of Wenzhou. The patients/participants provided their written informed consent to participate in this study.

## Author Contributions

XG conceived and designed the study. YZ, YC, WC, and JC obtained the data. YP analyzed and interpreted the data. LB and XG drafted the manuscript. All authors contributed to data analysis, drafting and revising the article, and approved the final version of the manuscript to be published. Moreover, they are accountable for all aspects of the work.

### Conflict of Interest Statement

The authors declare that the research was conducted in the absence of any commercial or financial relationships that could be construed as a potential conflict of interest.
