# Supplementary material for: Combination of Systemic Inflammation Response Index and Platelet-to-Lymphocyte Ratio as a Novel Prognostic Marker of Upper Tract Urothelial Carcinoma After Radical Nephroureterectomy
Source: Front Oncol. 2019 Sep 18;9:914. doi: 10.3389/fonc.2019.00914 (PMC6759944; doi:10.3389/fonc.2019.00914)
Supplement: Supplementary file 1 [file Data_Sheet_1.docx]

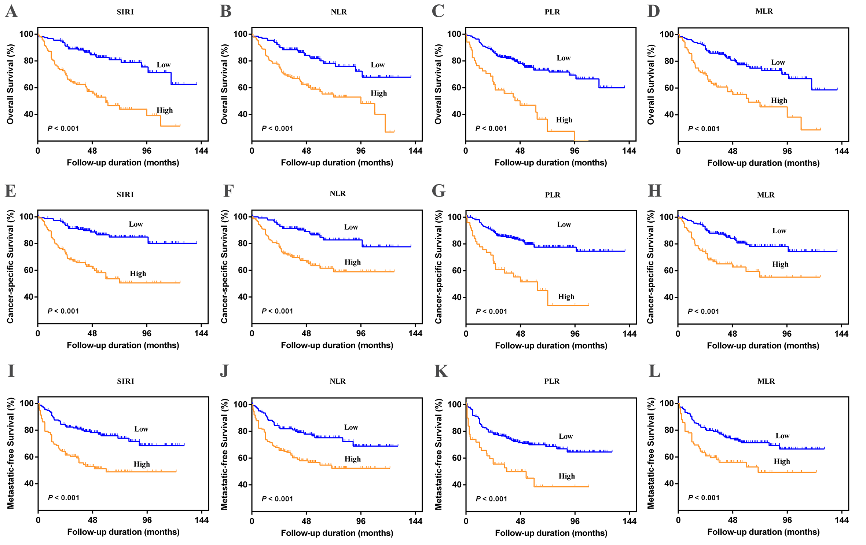


**Figure S1**. Kaplan-Meier curves for OS (A-D), CSS (E-H), and

MFS (I-L) in UTUC patients stratified by SIRI, NLR,

PLR, and MLR in the validation cohort.


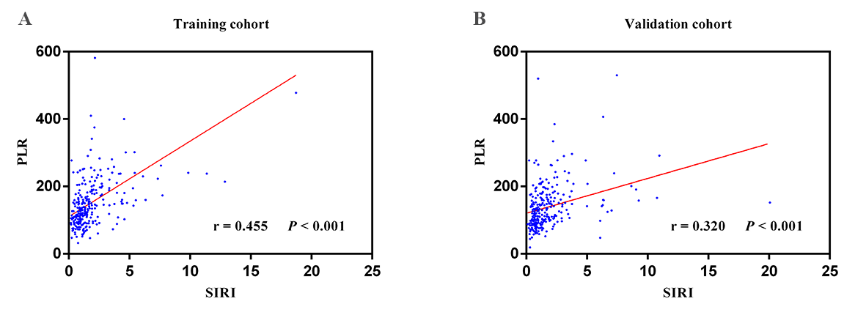


**Figure S2.** Correlation between SIRI and PLR in the training cohort (A) and the validation cohort (B).


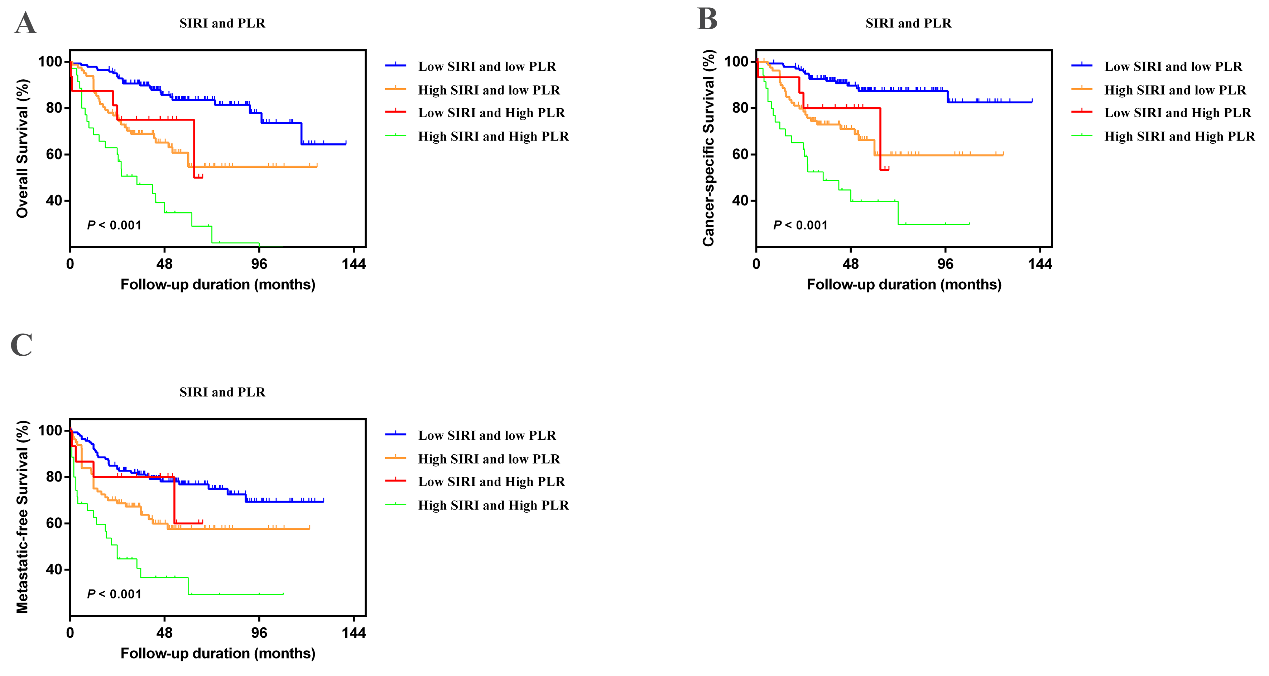


**Figure S3**. Kaplan-Meier analysis for OS (A), CSS (B), and MFS (C) in UTUC

patients based on SIRI-PLR in the validation cohort.


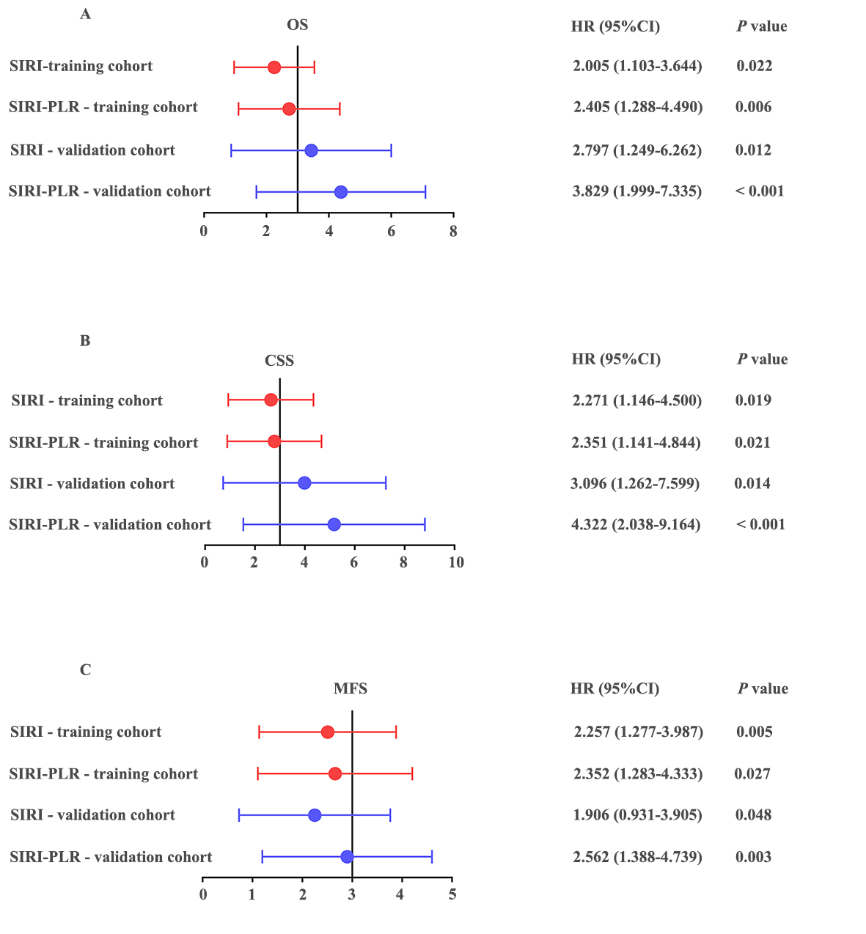


**Figure S4.** Comparison of HR values for OS (A), CSS (B), and MFS (C) between SIRI and SIRI-PLR in the training cohort and the validation cohort.


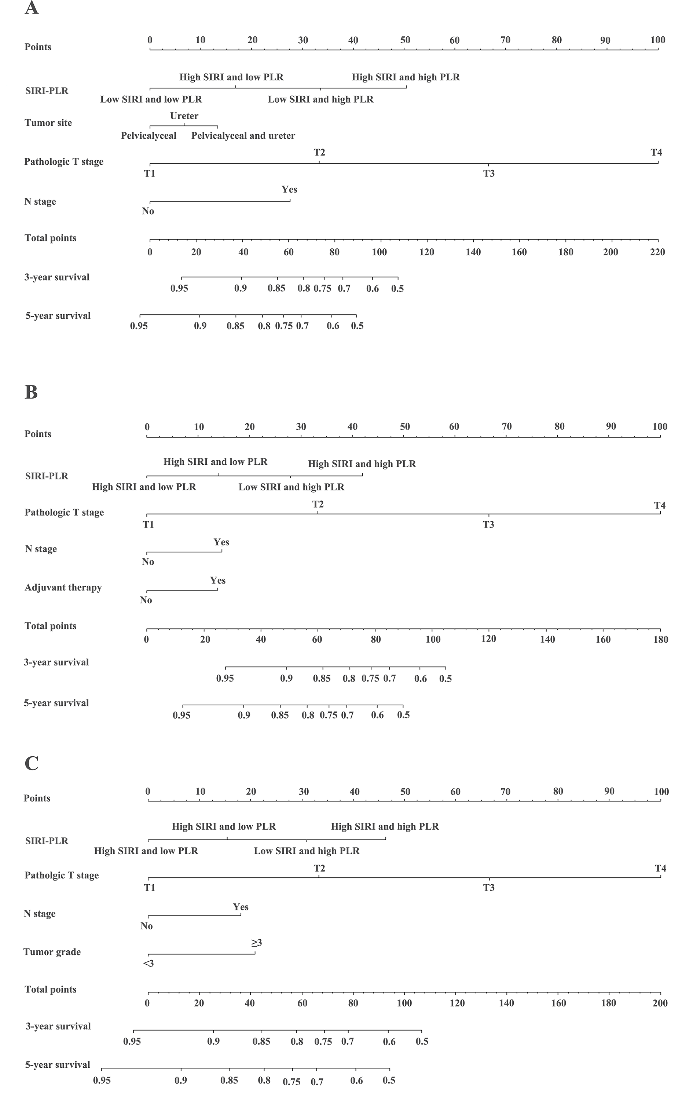


**Figure S5**. Postoperative nomograms to predict the probability of OS (A), CSS (B), and MFS (C) rates in patients with UTUC after surgery.


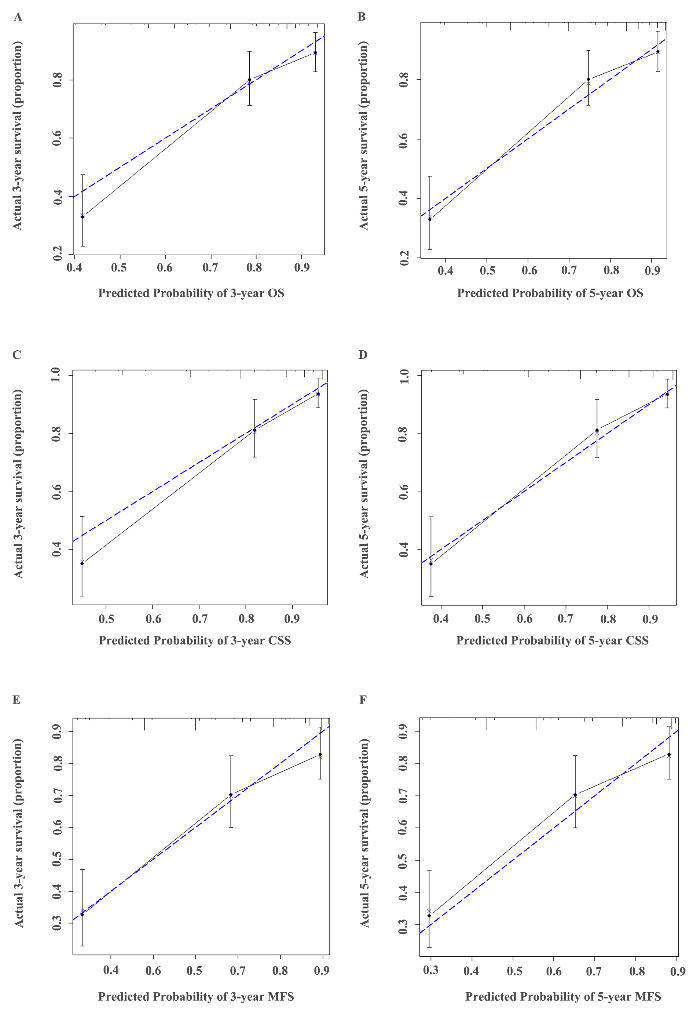


**Figure S6**. Calibration curve for predicting 3- and 5-year survival of OS (A and B), CSS (C and D), and MFS (E and F) in UTUC patients in the validation cohort.


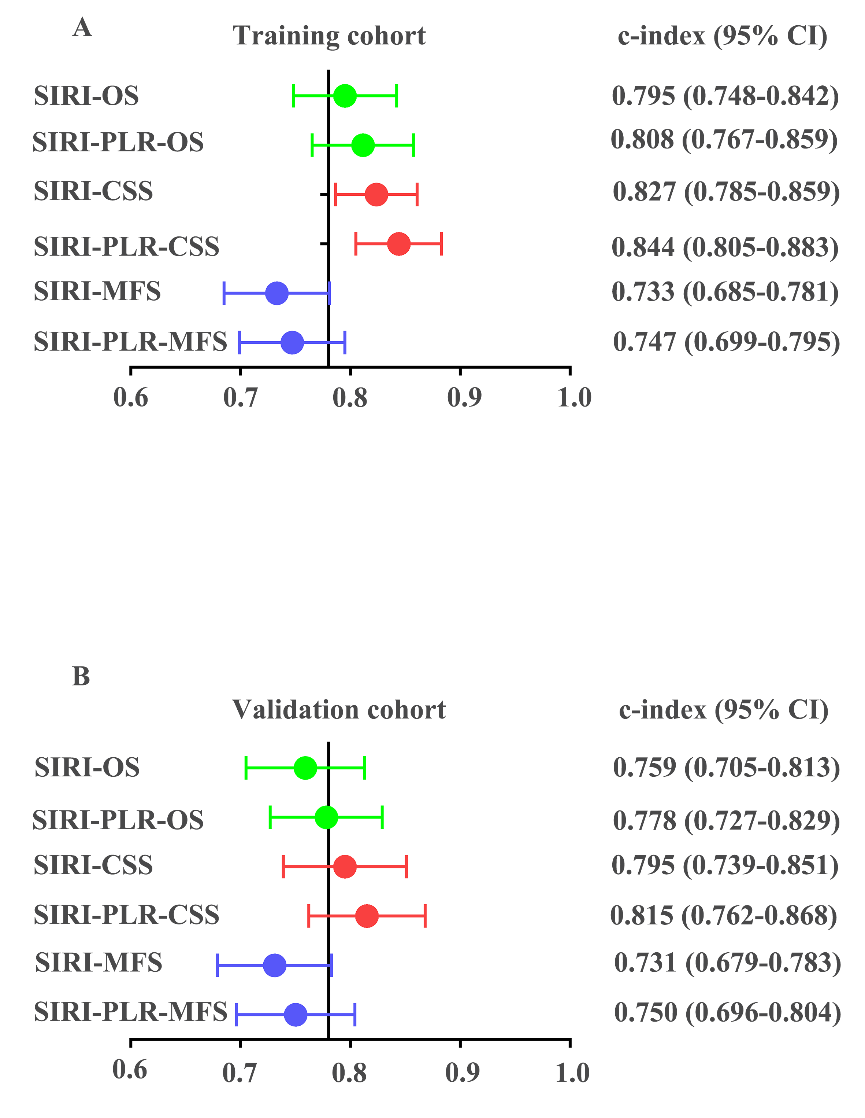


**Figure S7**. Comparison of c-index for OS, CSS, and MFS between SIRI and SIRI-PLR in the training cohort (A) and validation cohort (B).

Table S1 Univariate analysis of variables for the prediction of survival outcomes in validation cohort

| Variables | Overall survival | | | Cancer-specific survival | | | Metastasis-free survival | | |
| --- | --- | --- | --- | --- | --- | --- | --- | --- | --- |
|  | HR | 95%CI | *P* value | HR | 95%CI | *P* value | HR | 95%CI | *P* value |
| Gender (Male vs Female) | 1.401 | 0.868-2.262 | 0.167 | 1.514 | 0.872-2.629 | 0.141 | 1.269 | 0.804-2.002 | 0.306 |
| Age (>65 vs ≤65 years) | 1.327 | 0.846-2.080 | 0.218 | 1.093 | 0.665-1.799 | 0.725 | 1.087 | 0.711-1.660 | 0.701 |
| BMI (≥25 vs <25) | 0.762 | 0.474-1.223 | 0.260 | 0.738 | 0.429-1.269 | 0.272 | 0.832 | 0.530-1.306 | 0.424 |
| ASA grade (≥3 vs <3) | 1.063 | 0.608-1.858 | 0.831 | 0.787 | 0.390-1.590 | 0.505 | 0.934 | 0.528-1.653 | 0.815 |
| Hydronephrosis (Yes vs No) | 1.335 | 0.827-2.156 | 0.237 | 1.534 | 0.873-2.694 | 0.137 | 1.233 | 0.781-1.945 | 0.369 |
| Surgical approach (laparoscopic vs open) | 0.382 | 0.221-0.658 | **0.001*** | 0.386 | 0.206-0.722 | **0.003*** | 0.438 | 0.247-0.774 | **0.005*** |
| SIRI (≥1.36 vs<1.36) | 3.423 | 2.182-5.372 | **<0.001*** | 4.001 | 2.363-6.774 | **<0.001*** | 2.484 | 1.629-3.787 | **<0.001*** |
| NLR (≥2.53 vs<2.53) | 2.646 | 1.665-4.205 | **<0.001*** | 3.037 | 1.764-5.227 | **<0.001*** | 2.166 | 1.400-3.350 | **0.001*** |
| PLR (≥126.88 vs<126.88) | 3.147 | 1.992-4.972 | **<0.001*** | 3.472 | 2.096-5.752 | **<0.001*** | 2.413 | 1.527-3.815 | **<0.001*** |
| MLR (≥0.35 vs<0.35) | 2.705 | 1.764-4.149 | **<0.001*** | 2.606 | 1.605-4.232 | **<0.001*** | 2.045 | 1.346-3.106 | **0.001*** |
| Anemia (Yes vs No) | 2.705 | 1.764-4.149 | **<0.001*** | 2.034 | 1.248-3.316 | **0.004*** | 1.852 | 1.211-2.831 | **0.004*** |
| Hypoproteinemia (Yes vs No) | 1.889 | 1.000-3.566 | 0.050* | 1.772 | 0.845-3.716 | 0.130 | 1.238 | 0.599-2.560 | 0.564 |
| CKD stage |  |  |  |  |  |  |  |  |  |
| CKD 1 | 1.000 | Reference | 1.000 | 1.000 | Reference | 1.000 | 1.000 | Reference | 1.000 |
| CKD 2-3 | 0.857 | 0.511-1.437 | 0.558 | 0.689 | 0.398-1.191 | 0.182 | 0.760 | 0.474-1.216 | 0.252 |
| CKD 4-5 | 1.016 | 0.405-2.550 | 0.973 | 0.539 | 0.159-1.833 | 0.322 | 0.413 | 0.124-1.375 | 0.150 |
| Tumor size (≥3 vs<3) | 1.662 | 1.078-2.561 | **0.021*** | 1.822 | 1.121-2.962 | **0.015*** | 1.473 | 0.965-2.247 | 0.073 |
| Tumor site |  |  |  |  |  |  |  |  |  |
| Pelvicalyceal | 1.000 | Reference | 1.000 | 1.000 | Reference | 1.000 | 1.000 | Reference | 1.000 |
| Ureter | 0.738 | 0.462-1.179 | 0.204 | 0.749 | 0.442-1.271 | 0.284 | 0.814 | 0.523-1.266 | 0.361 |
| Both | 2.259 | 1.138-4.483 | **0.020*** | 1.944 | 0.866-4.366 | 0.107 | 1.390 | 0.630-3.065 | 0.414 |
| Multifocality (Yes vs No) | 1.472 | 0.927-2.336 | 0.101 | 1.367 | 0.802-2.330 | 0.251 | 1.159 | 0.722-1.861 | 0.542 |
| Pathologic T stage |  |  |  |  |  |  |  |  |  |
| pT1 | 1.000 | Reference | 1.000 | 1.000 | Reference | 1.000 | 1.000 | Reference | 1.000 |
| pT2 vs pT1 | 3.052 | 1.326-7.023 | **0.009*** | 2.504 | 0.839-7.474 | 0.100 | 1.982 | 0.937-4.190 | 0.073 |
| pT3 vs pT1 | 8.170 | 3.839-17.388 | **<0.001*** | 10.476 | 4.117-26.659 | **<0.001*** | 5.587 | 2.955-10.564 | **<0.001*** |
| pT4 vs pT1 | 18.827 | 7.714-45.949 | **<0.001*** | 28.605 | 10.116-80.885 | **<0.001*** | 11.936 | 5.490-25.953 | **<0.001*** |
| N stage (N1 vs N0) | 4.410 | 2.379-8.177 | **<0.001*** | 4.089 | 2.016-8.297 | **<0.001*** | 2.866 | 1.481-5.545 | **0.002*** |
| Tumor grade (≥3 vs <3) | 3.357 | 1.840-6.125 | **<0.001*** | 3.969 | 1.886-8.352 | **<0.001*** | 2.941 | 1.655-5.225 | **<0.001*** |
| LVI (Yes vs No) | 2.972 | 1.786-4.949 | **<0.001*** | 3.141 | 1.800-5.480 | **<0.001*** | 2.629 | 1.607-4.300 | **<0.001*** |
| Adjuvant therapy (Yes vs No) | 1.675 | 1.072-2.617 | **0.024*** | 2.016 | 1.228-3.307 | **0.006*** | 1.609 | 1.041-2.487 | **0.032*** |

Note: *statistically significant

Table S2 Multivariate analysis of variables for the prediction of survival outcomes in validation cohort

| Variables | Overall survival | | | Cancer-specific survival | | | Metastasis-free survival | | |
| --- | --- | --- | --- | --- | --- | --- | --- | --- | --- |
|  | HR | 95%CI | *P* value | HR | 95%CI | *P* value | HR | 95%CI | *P* value |
| Surgical approach (laparoscopic vs open) | 0.795 | 0.419-1.507 | 0.482 | 0.697 | 0.338-1.436 | 0.328 | 0.648 | 0.336-1.251 | 0.196 |
| SIRI (≥1.36 vs<1.36) | 2.797 | 1.249-6.262 | **0.012*** | 3.096 | 1.262-7.599 | **0.014*** | 1.906 | 0.931-3.905 | **0.048*** |
| NLR (≥2.53 vs<2.53) | 0.646 | 0.295-1.414 | 0.274 | 0.695 | 0.285-1.694 | 0.424 | 0.865 | 0.431-1.733 | 0.682 |
| PLR (≥126.88 vs<126.88) | 1.839 | 1.040-3.253 | **0.036*** | 1.951 | 1.047-3.634 | **0.035*** | 1.381 | 0.794-2.402 | 0.252 |
| MLR (≥0.35 vs<0.35) | 1.079 | 0.595-1.955 | 0.802 | 0.960 | 0.508-1.812 | 0.899 | 1.068 | 0.604-1.887 | 0.821 |
| Anemia (Yes vs No) | 1.558 | 0.965-2.514 | 0.070 | 1.460 | 0.837-2.546 | 0.182 | 1.443 | 0.906-2.298 | 0.123 |
| Hypoproteinemia (Yes vs No) | 1.212 | 0.592-2.480 | 0.599 |  | - |  |  | - |  |
| Tumor size (≥3 vs<3) | 1.082 | 0.665-1.762 | 0.750 | 0.938 | 0.542-1.632 | 0.818 | 0.978 | 0.617-1.550 | 0.924 |
| Tumor site |  |  |  |  |  |  |  |  |  |
| Pelvicalyceal | 1.000 | Reference | 1.000 |  | - |  |  | - |  |
| Ureter | 0.852 | 0.504-1.441 | 0.550 |  |  |  |  |  |  |
| Both | 2.753 | 1.295-5.853 | **0.008*** |  |  |  |  |  |  |
| Pathologic T stage |  |  |  |  |  |  |  |  |  |
| pT1 | 1.000 | Reference | 1.000 | 1.000 | Reference | 1.000 | 1.000 | Reference | 1.000 |
| pT2 vs pT1 | 3.414 | 1.420-8.206 | **0.006*** | 2.261 | 0.753-6.790 | 0.146 | 1.833 | 0.863-3.891 | 0.115 |
| pT3 vs pT1 | 6.296 | 2.803-14.143 | **<0.001*** | 6.598 | 2.503-17.393 | **<0.001*** | 4.113 | 2.110-8.019 | **<0.001*** |
| pT4 vs pT1 | 15.865 | 5.899-42.673 | **<0.001*** | 22.792 | 7.371-70.472 | **<0.001*** | 10.278 | 4.337-24.357 | **<0.001*** |
| N stage (N1 vs N0) | 1.780 | 0.641-4.940 | **0.028*** | 1.573 | 0.526-4.707 | **0.018*** | 1.312 | 0.498-3.456 | **0.033*** |
| Tumor grade (≥3 vs <3) | 1.596 | 0.805-3.166 | 0.181 | 1.809 | 0.786-4.164 | 0.164 | 1.660 | 0.875-3.148 | 0.121 |
| LVI (Yes vs No) | 1.849 | 1.379-2.900 | 0.691 | 1.780 | 1.343-2.776 | 0.554 | 1.883 | 1.429-2.815 | 0.735 |
| Adjuvant therapy (Yes vs No) | 1.373 | 0.830-2.271 | 0.217 | 1.753 | 1.006-3.053 | **0.047*** | 1.556 | 0.970-2.494 | 0.067 |

Note: *statistically significant

Table S3 Multivariate analysis of variables for the prediction of survival outcomes in validation cohort when interrelated SIRI and PLR are combined.

| Variables | Overall survival | | | Cancer-specific survival | | | Metastasis-free survival | | |
| --- | --- | --- | --- | --- | --- | --- | --- | --- | --- |
|  | HR | 95%CI | *P* value | HR | 95%CI | *P* value | HR | 95%CI | *P* value |
| Surgical approach (laparoscopic vs open) | 0.781 | 0.410-1.486 | 0.451 | 0.693 | 0.339-1.415 | 0.314 | 0.596 | 0.312-1.141 | 0.119 |
| SIRI-PLR |  |  |  |  |  |  |  |  |  |
| Low SIRI + low PLR | 1.000 | Reference | 1.000 | 1.000 | Reference | 1.000 | 1.000 | Reference | 1.000 |
| High SIRI+low PLR vs low SIRI + low PLR | 2.100 | 1.181-3.732 | **0.011*** | 2.258 | 1.165-4.377 | **0.016*** | 1.473 | 0.877-2.476 | 0.143 |
| Low SIRI+high PLR vs low SIRI + low PLR | 1.533 | 0.525-4.473 | 0.435 | 1.537 | 0.463-5.096 | 0.482 | 0.727 | 0.241-2.189 | 0.570 |
| High SIRI+high PLR vs low SIRI + low PLR | 3.829 | 1.999-7.335 | **<0.001*** | 4.322 | 2.038-9.164 | **<0.001*** | 2.565 | 1.388-4.739 | **0.003*** |
| Anemia (Yes vs No) | 1.586 | 0.989-2.544 | 0.056 | 1.476 | 0.875-2.488 | 0.144 | 1.423 | 0.909-2.228 | 0.123 |
| Hypoproteinemia (Yes vs No) | 1.168 | 0.574-2.376 | 0.668 |  | - |  |  | - |  |
| Tumor size (≥3 vs<3) | 1.067 | 0.656-1.736 | 0.793 | 0.933 | 0.541-1.608 | 0.802 | 0.979 | 0.618-1.549 | 0.927 |
| Tumor site |  |  |  |  |  |  |  |  |  |
| Pelvicalyceal | 1.000 | Reference | 1.000 |  | - |  |  | - |  |
| Ureter | 0.829 | 0.493-1.395 | 0.481 |  |  |  |  |  |  |
| Both | 2.712 | 1.277-5.761 | **0.009*** |  |  |  |  |  |  |
| Pathologic T stage |  |  |  |  |  |  |  |  |  |
| pT1 | 1.000 | Reference | 1.000 | 1.000 | Reference | 1.000 | 1.000 | Reference | 1.000 |
| pT2 vs pT1 | 3.363 | 1.401-8.071 | **0.007*** | 2.234 | 0.744-6.704 | 0.152 | 1.794 | 0.846-3.805 | 0.128 |
| pT3 vs pT1 | 6.424 | 2.858-14.438 | **<0.001*** | 6.737 | 2.566-17.689 | **<0.001*** | 4.043 | 2.085-7.839 | **<0.001*** |
| pT4 vs pT1 | 15.832 | 5.867-42.722 | **<0.001*** | 22.742 | 7.412-69.777 | **<0.001*** | 9.656 | 4.112-22.674 | **<0.001*** |
| N stage (N1 vs N0) | 1.693 | 0.588-4.879 | **0.029*** | 1.487 | 0.466-4.750 | **0.003*** | 1.015 | 0.369-2.790 | **0.027*** |
| Tumor grade (≥3 vs <3) | 1.521 | 0.766-3.022 | 0.231 | 1.743 | 0.758-4.010 | 0.191 | 1.690 | 0.896-3.189 | 0.105 |
| LVI (Yes vs No) | 1.850 | 1.374-1.932 | 0.699 | 0.793 | 0.346-1.816 | 0.583 | 1.023 | 0.500-2.091 | 0.951 |
| Adjuvant therapy (Yes vs No) | 1.349 | 0.818-2.223 | 0.240 | 1.763 | 1.018-3.054 | **0.043*** | 1.561 | 0.976-2.497 | 0.063 |

Note: *statistically significant
